# Strategies for Pathogen Biocontrol Using Lactic Acid Bacteria and Their Metabolites: A Focus on Meat Ecosystems and Industrial Environments

**DOI:** 10.3390/microorganisms5030038

**Published:** 2017-07-11

**Authors:** Patricia Castellano, Mariana Pérez Ibarreche, Mariana Blanco Massani, Cecilia Fontana, Graciela M. Vignolo

**Affiliations:** 1 Centro de Referencia para Lactobacilos (CERELA), CONICET, Chacabuco 145, Tucumán T4000ILC, Argentina; patricia@cerela.org.ar (P.C.); marianapi@outlook.com.ar (M.P.I.); 2 INTI-Plásticos, Gral Paz 5445 e/Constituyentes y Albarelos, B1650KNA Gral, San Martín, Buenos Aires, Argentina; marianablanco.m@gmail.com; 3 Instituto Nacional de Tecnología Agropecuaria INTA-EEA, Ruta Provincial 301 Km 32, Famaillá 4132, Tucumán, Argentina; fontana.cecilia@inta.gob.ar

**Keywords:** biocontrol, lactic acid bacteria, bacteriocins, meat products

## Abstract

The globalization of trade and lifestyle ensure that the factors responsible for the emergence of diseases are more present than ever. Despite biotechnology advancements, meat-based foods are still under scrutiny because of the presence of pathogens, which causes a loss of consumer confidence and consequently a fall in demand. In this context, Lactic Acid Bacteria (LAB) as GRAS organisms offer an alternative for developing pathogen-free foods, particularly avoiding *Listeria monocytogenes*, with minimal processing and fewer additives while maintaining the foods’ sensorial characteristics. The use of LAB strains, enabling us to produce antimicrobial peptides (bacteriocins) in addition to lactic acid, with an impact on quality and safety during fermentation, processing, and/or storage of meat and ready-to-eat (RTE) meat products, constitutes a promising tool. A number of bacteriocin-based strategies including the use of bioprotective cultures, purified and/or semi-purified bacteriocins as well as their inclusion in varied packaging materials under different storage conditions, have been investigated. The application of bacteriocins as part of hurdle technology using non-thermal technologies was explored for the preservation of RTE meat products. Likewise, considering that food contamination with *L. monocytogenes* is a consequence of the post-processing manipulation of RTE foods, the role of bacteriocinogenic LAB in the control of biofilms formed on industrial surfaces is also discussed.

## 1. Introduction

Trends in food consumption, technology, and trade likely impact on microbial food safety. Modern life conditions, related to or in consequence of globalization, contribute to the major incidence of food disease outbreaks. Infectious intestinal diseases caused by bacteria, viruses, or parasites continue to be a major source of public health concern and social and economic costs worldwide. Developed countries have for a long time used surveillance systems for food safety problems, allowing estimations of the impact of disease and the risks associated with consuming different foods. Summary report on trends and sources of zoonoses, zoonotic agents, and food-borne outbreaks in the European Union (EU) in 2015 indicated that the occurrence of *Campylobacter* remained high in broiler meat, followed by the presence of *Salmonella* in poultry and turkey meat, with yersiniosis being the third most commonly reported zoonosis in the EU. With a lower impact, listeriosis was reported from RTE foods, mainly from fishery products and soft cheeses, while the highest proportion of Shiga toxin *Escherichia (E.) coli* (STEC)-positive food samples was detected in meat from ruminants [1]. In the USA, the reported foodborne outbreaks predominantly involved the presence of *Salmonella* in raw vegetables, chicken/eggs, pork, and tuna, followed by *Listeria (L.). monocytogenes* from dairy products (raw milk, soft cheeses, ice cream), while STEC was mostly present in raw beef, chicken salads, and raw vegetables [2].

On this basis, the need for solutions regarding the hygienic quality of foods has been stated. Modern consumer trends and food legislation have assumed the successful attainment of food preservation to be more than a challenge. Since consumers demand high-quality, preservative-free, safe, and minimally processed foods with extended shelf-life, and legislation has restricted the use and permitted level of some of the currently approved preservatives in different foods, both consumer and legislators need to call for innovative approaches to preserving foods. For many centuries, microbial antagonism has been used in food processing to improve food safety. An understanding of the mode of action of microbial antagonisms has been gained in recent years, increasing attention to it as a means of naturally controlling the shelf-life and safety of foods and feed; feed is now recognized as the first step in the food chain as animal welfare and feed can influence food safety. Biological preservation to ensure the hygienic quality of food has become a promising tool [3]. In this context, the primary role of lactic acid bacteria (LAB) during the fermentation of food substrates was their acidifying ability to produce unique flavor, aroma, and texture characteristics as well as to control spoilage and pathogenic organisms. Their natural presence in raw materials and fermented foods granted them GRAS status due to a general recognition of safety, based on experience from common use in food over the centuries. Although knowledge on LAB antimicrobial peptides, named bacteriocins, has dramatically increased during the last 30 years, their application as preservatives has not met with equal success; restrictive legislation concerning food additives, the limited inhibition spectrum and food constituents’ effects on efficiency could explain the lack of industrial applications of bacteriocins. Nisin, a bacteriocin produced by *Lactococcus (Lc) lactis,* is the only bacteriocin approved by the Food and Drug Administration (FDA) for use in more than 50 countries. The introduction of bacteriocins in the food matrix by in situ production using live LAB, the so-called bioprotective cultures, is an alternative approach.

## 2. Antimicrobial Potential of LAB

Because LAB have been part of raw material and fermented foods since ancient times, their association with health has grown among consumers and they have become increasingly popular. The main reason for this is the health-friendly reputation of natural preservation methods compared to chemical or physicochemical treatments. In addition, shelf-life extension of foods with simple technological procedures accessible to smaller economies, the possibility of solving emerging issues in the food chain, the presence of emergent pathogens, and the improvement of animal and human health by natural means must also be taken into account. Thus, because of increased consumer demand for higher quality and natural foods, and the strict government regulations to guarantee food safety, food producers face conflicting interests. Based on the wide spectrum of produced antimicrobial compounds, LAB can be exploited as microbial cell factories and used in several applications such as biopreservation, shelf-life extension, fermentation biocontrol, human and veterinary medicine, and agriculture [4,5].

Organic acid production by LAB and a decrease in pH constitute the main mechanisms of biopreservation in fermented foods. However, LAB strains are able to produce other antimicrobial substances such as low molecular weight metabolites (reuterin, reutericyclin, diacetyl, fatty acids), hydrogen peroxide, antifungal compounds (propionate, phenyl-lactate, hydroxyphenyl-lactate and 3-hydroxy fatty acids), and bacteriocins that may be exploited in the biopreservation of foods. After the breakthrough in the processing of fermented foods that was the deliberate addition of LAB as a starter culture, the use of a novel generation of starter cultures offering functionalities beyond acidification has recently been explored. LABs are capable of inhibiting different microorganisms in food, displaying crucial antimicrobial effects with a strong impact on preservation and safety. In addition, since bacteriocins are able to kill bacteria by disruption of membrane integrity, they are thought to be less likely to induce resistance; small peptides produced by LAB are considered a potential solution to the growing problem of resistance to conventional antibiotics [4,5,6]. Despite the broad field of application, one of the main constraints of many bacteriocins is their narrow spectrum of inhibition. Although most bacteriocins are only active against Gram-positive bacteria, some LAB bacteriocins were described to exhibit activity against Gram-negative organisms of concern in foods and human/animal GIT [7]. Moreover, as most pathogens that cause foodborne diseases are Gram-negative bacteria, strategies for the use of bacteriocins in these microorganisms have been evaluated. Bacteriocins have a greater opportunity to target Gram-negative pathogens if the outer membrane has been destabilized by the presence of another hurdle. Many potential hurdles for food preservation have been described, such as mild treatments using organic acids, chelating agents, and plant essential oils or by physical treatments such as heating, freezing, high-pressure processing, or pulsed electrical fields [8]. Hurdle technology is especially attractive in exploiting bacteriocins, as some peptides have demonstrated additive or synergistic effects when used in combination with other compounds or physical treatments providing an approach to minimize the development of resistant strains [9]. Organic acids can work as enhancers of bacteriocins’ effect as the increase of peptides’ net charge at low pH facilitates bacteriocins’ translocation through the cell wall, as well as their solubility [10]. Recently, the availability of bacterial genomes provided valuable information on the bacteriocinogenic potential of LAB, shedding light on the global response of different ecosystems to bacteriocins as well as the development of adaptation or resistance [11,12].

### Bacteriocins from LAB

Ribosomally synthesized antimicrobial peptides, commonly referred to as bacteriocins, have been found to be widespread amongst food-grade bacteria such as LABs, which are highly specific against closely related bacteria. Although various Gram-positive bacteria are capable of producing bacteriocins, LAB bacteriocins are by far the most studied due to the food origin and GRAS status of producer strains; therefore, bacteriocins are also expected to be safe [13]. In addition to being nontoxic, these peptides are innocuous, having no or little influence on the gut microbiota since they can be digested by proteases. Differences in molecular mass, posttranslational modifications, presence of modified amino acids, chemical structure as well as mode of action allow for dividing LAB bacteriocins into different classes. Up to 2015, 185 LAB bacteriocins had been isolated and only 53% of them were well characterized and sequenced at the protein/DNA level [14]. Several approaches have been proposed to classify bacteriocins [15,16]; the main distinction was made between lantibiotics (Class Ia), which undergo post-translational modifications, and non-modified peptides (Class II).

**Class I bacteriocins** are a group of small peptides (<5 kDa) possessing unusual post-translational modified residues such as lanthionine or 3-methyllanthionine, which result in characteristic inner-molecular-ring structures. Nisin is the prototype bacteriocin from **Class Ia** and is produced by many strains of *Lactococcus (Lc.) lactis*, a species used in cheese manufacture. It has a broad antimicrobial spectrum against a wide range of Gram-positive genera (staphylococci, streptococci, *Listeria* spp., bacilli, and enterococci). Nisin A has been used in the food industry as a biopreservative for more than 50 years. The antimicrobial effect of nisin is caused by its interaction with the precursor of peptidoglycan and lipid II on the bacterial cell wall. Upon binding to the target molecule, the nisin–lipid II complex inserts itself into the bacterial cell membrane, forming pores creating leakage of essential cellular components that conduct the cell to its death [16]. To date, several natural nisin variants have been discovered, including nisin A, Z, F, and Q, which have been isolated from lactococci, nisin H from *Streptococcus (Str.) hyointestinalis*, nisin U and U2, from *Str. uberis*, and nisin P, which is encoded on nisin operons from both, *Str. gallolyticus* subsp. *pasteurianus* and *Str. suis* [17]. Alignment of the amino acid sequences of different nisins shows an increasing number of amino acid changes from the prototypical lactococcal nisin A, ranging from 1 amino acid position for nisin Z up to 13 positions for streptococcal nisin P [14,18].

**Class II bacteriocins** involve a large and heterogeneous group of small (<10 kDa) heat-stable peptides characterized by their non-modified nature [16,18]. Based on structural and functional characteristics, this bacteriocin class may be divided into several groups. **Subclass IIa** or pediocin-like bacteriocins can be considered the major subgroup, with more than 50 characterized members [19]. These peptides have narrow inhibitory spectra but are known for their strong anti-listerial activity, and have been extensively studied with respect to genetics, structure, and mode of action. Moreover, many species within the genus *Enterococcus* are able to produce pediocin-like bacteriocins [16]. Pediocin PA-1 is a broad-spectrum LAB bacteriocin that shows a particularly strong activity against *L. monocytogenes*, a foodborne pathogen of special concern to the food industry. This antimicrobial peptide is the most extensively studied class IIa bacteriocin, and has been sufficiently well characterized to be used as a food biopreservative [19]. Based on differences of C-terminal Subclass IIa, bacteriocins were separated into three subgroups [20]; examples of bacteriocins produced by meat borne LAB are pediocin PA-1/AcH, sakacin P, and enterocin A (subgroup I); leucocin A and sakacin G (subgroup II); and curvacin A, carnobacteriocin B2, and enterocin P (subgroup III).

**Subclass IIb** contains two-peptide bacteriocins whose activity is dependent on the combined action of two non-identical peptide moieties, while each peptide individually exerts low or no antimicrobial activity [21]. Lactocin 705, produced by *Lactobacillus (L.) curvatus* CRL705, was the first two-peptide bacteriocin reported from a meat-borne LAB strain, which showed inhibitory activity against other LAB and *Brochothrix (Br.) thermosphacta* [22,23]. In addition, three novel bacteriocins belonging to class II were recently reported, such as enterocin NKR-5-3C a pediocin-like bacteriocin (produced by *Enterococcus (E.) faecium* NKR-5-3 from Thai fermented fish) as well as lactococcin Q (*Lc. lactis* QU4 from corn) and enterocin X (*E. faecium* KU-B5 from Thai sugar apples), both belonging to two-peptide bacteriocins [14,18].

**Class IIc** are circular bacteriocins characterized by a head-to-tail covalent bond forming a circular structure. A relative broad inhibitory spectrum and resistance to heat, extreme pH, and even many proteases was described for these bacteriocins [16]. Several circular bacteriocins composed of 58 to 70 amino acid residues have been identified [14]. Among those produced by meat-borne LAB strains, enterocin AS-48 (produced by *Enterococcus* species from different origins), carnocyclin A (*Carnobacterium (C.) maltaromaticum* UAL307 from pork), garvicin ML (*Lc. garvieae* DCC43 from mallard ducks) were described [24]. On the other hand, **Class IId** involves the remaining well-characterized bacteriocins, including other non-pediocin-like linear one-peptide bacteriocins, sec-dependent, double-glycine leader, and leaderless bacteriocins [16]. A number of leaderless bacteriocins have recently been reported, such as lacticin Q and its homologue lacticin Z, which were produced by *Lc. lactis* QU5 (from corn) and *Lc. lactis* QU14 (from horse intestines), respectively [18]. In addition, *Weissella (W.) hellenica* QU 13, isolated from Japanese pickles, was recently found to produce two novel leaderless bacteriocins, weissellicins Y and M [18]. LAB bacteriocins are diverse in terms of molecular mass, posttranslational modification, presence of modified amino acids, chemical structure, and mode of action. Up to 2015, 185 LAB bacteriocins have been isolated and reported [14]. In particular, the occurrence of antilisterial structural bacteriocins genes in meat-borne lactic acid bacteria has been recently reported; the potential of *L. sakei* and *E. faecium* to be used as bioprotective cultures provide and additional hurdle to enhance the control of *L. monocytogenes* in raw meat and meat products [12].

## 3. Meat Ecosystems as Pathogens Source

Many foodborne diseases are associated with consumption of meat and poultry. Although muscle of healthy animals are regarded as sterile, meat as well as their fermented and processed products provide an excellent environment for the growth of pathogenic and spoilage microorganisms. The public health risks associated with meat and poultry products, the scientific community’s understanding of their causes and human health impacts, and the various regulatory responses to them have changed considerably over time. As previously reported [25,26], some pathogens were not previously known (new pathogens), others have newly arisen as foodborne (emerging pathogens), and others have become more potent or associated with other products (evolving pathogens). The major sources of the initial microbiota found on meat carcasses are the slaughter animals themselves, the process workers, and the processing environment. The animal sources of contaminating microorganisms include external body surfaces (skin, hide, fleece, feathers, feet, and hooves) and the gastrointestinal and respiratory tracts [25,26,27]. The microbiological quality of a dressed carcass is determined by a complex interaction between the microbiota carried by the live animal and the hygienic efficiency of the slaughter and dressing process. A substantial proportion of all emerging infections is associated with farm animals and meat. The most prevalent and serious emerging pathogens of meat, poultry, and derived products are *Campylobacter (C.) jejuni*, *Salmonella (S.) typhimurium*, *E. coli* O157:H7, and other enterohemorrhagic *E. coli* (EHEC), *L. monocytogenes*, *Staphylococcus (S.) aureus*, *Yersinia (Y.) enterocolitica* and the potential for *Clostridium (Cl.) botulinum* in cured hams and sausages [26,28]. Although pork is less associated with foodborne illness than other meat sources, it remains significant due to its largest consumption in a variety of products [27]. Unless subsequent processing includes antimicrobial treatments, microbial numbers increase progressively with further processing, at first because of additional contamination associated with handling, and later because of microbial growth. Pathogenic microorganisms, particularly those of animal origin, can also be expected to be found in the saprophyte-dominated initial microflora, although, except in unusual circumstances, their numbers are generally low. Beef, pork, poultry, and other animal products are estimated to be among the most important food vehicles for all of these pathogens, which are associated with direct or indirect fecal contamination of carcasses, with the exception of *S. aureus*, which is a result of contamination from food handlers. Other pathogens such as *L. monocytogenes* may be animal-associated, but are mostly found in processing environments as a consequence of post-processing operations.

## 4. Antimicrobial Interventions

The different muscle-food ecosystems offer a great variety of scenarios in which pathogenic and spoilage microorganisms may proliferate, this being dependent on the raw materials, processing conditions, distribution, and consumption. Careful tests against specific target bacteria in the type of food in which they will be applied are required to assure bacteriocins’ effectiveness. Antagonistic microorganisms or their metabolic products being used to control undesirable (pathogen and contaminant) organisms to extend shelf-life and enhance food safety without altering the sensory characteristics of the food product is referred to as bioprotection. Two main approaches are commonly used in the application of bacteriocins for food biopreservation: (i) ex situ production, involving the addition of either purified or semi-purified bacteriocins as food preservatives, which is dependent on legal approval/regulations or the use of a previously fermented product with a bacteriocin-producing LAB strain as an ingredient in food, and (ii) in situ production, involving the inoculation of food with bacteriocinogenic LAB strains, the ability of these strains to grow and produce bacteriocins in the products being crucial for its successful application. In the first case, bacteriocins recovered after cultivation of the producer strain in a food-grade substrate are added as partially purified or purified concentrates and will require approval as preservatives. As mentioned before, nisin is the only bacteriocin licensed as a food preservative in over 50 countries. The joint FAO/WHO Expert Committee on Food Additives established the daily permitted doses [29]; however, maximum limits of nisin are variable among countries. Dairy products (especially cheeses), meat and meat products including poultry and game, as well as canned foods are the products for which nisin has been regulated [2]. In addition, concentrated supernatants of a bacteriocin LAB producer (*P. acidilactici*) culture such as ALTA™2341 (Quest International, Sarasota, FL, USA), MicroGARD^®^™ (DuPont-Danisco, Thomson, IL, USA), and SAFEPRO^®^ (CHr. Hansen, Hørsholm, Denmark) are available commercially. A comprehensive strategy for foodborne pathogen control should be based on an integrated approach, which involves application of interventions at pre-harvest (in the field), post-harvest (at slaughter), processing (industrial plant), storage, distribution and merchandizing (retailing), food service, and consumption at home. Potential interventions include animal management strategies, competitive enhancement strategies, and direct anti-pathogen strategies.

### 4.1. Antimicrobial Interventions during Meat Conditioning and Raw Meats

The application of antimicrobial interventions to improve the microbiological quality of meat should start at pre-harvest or in the field. On-farm pathogen reduction programs contribute to controlling food safety problems, decreasing the probability of pathogen presence and reducing environmental pollution. Pre-harvest pathogen control interventions that have been proposed or explored include diet manipulation, feed additives/supplements use, antibiotics, bacteriophage therapy, vaccines administration/immunization, competitive exclusion, and prebiotics/probiotics administration; proper animal management practices such as pens handling, clean feed, chlorinated water, and non-stressful lairage and transportation to slaughter facilities are also important issues [25,27,30]. It is generally agreed that pre-harvest interventions may be needed to make significant additional progress, this being especially true considering that animal excretions contribute the majority of the pathogens that eventually contaminate beef products [30]. The parallel and/or simultaneous application of pre-slaughter strategies has the potential to synergistically reduce the incidence of human food-borne illnesses by multiple hurdles applications, thus preventing the entry of pathogens into the food chain. Several experimental pre-harvest interventions have been demonstrated to be effective for reducing the shedding of pathogens in cattle. Irrespective of pathogen control procedures applied to animals pre-harvest, it is also important to apply proper animal manure treatment and disposal procedures in order to limit the spread of pathogens in the environment, water, and other food crops [25].

Meat industry efforts at contamination control during meat conditioning will assure safe raw fresh meat in compliance with regulatory requirements. During harvest, activities should be designed to minimize the introduction of additional contamination as well as to implement sanitization interventions to reduce existing risks. Although the prevalence of pathogens in the beef chain varies considerably from survey to survey, contamination sources involve feces, ingest, hides, lymph nodes, and/or intestines of animals themselves as well as other factors such as the season and cattle rearing method [31]. Thus, decontamination with multiple interventions involves the sequential application of animal hide cleaning, chemical dehairing, hide removal, knife-trimming and/or steam-vacuuming of visibly soiled carcass spots, pre-evisceration carcass washing, evisceration, final carcass spray-cleaning, and chilling of eviscerated carcasses [25]. Contamination of carcasses at slaughter can occur in two ways, through previously infected live animal and/or cross-contamination of the carcass from the slaughter environment. Evisceration provides an opportunity for carcass contamination to arise, but any contamination that occurs is likely reduced by chilling. Even so, different combinations of interventions were tested for the decontamination of animal carcasses. Acids antimicrobials such as acetic, citric, and lactic acids and their salts are widely used in the USA and Canada for carcass decontamination [30,32]. Table 1 shows antimicrobial interventions of LAB and their metabolites against pathogenic microorganisms as well as bacteriophages on different meat products [33,34,35,36]. The bacteriocins produced by LAB have been used as antimicrobial interventions in beef and poultry carcasses and raw meat with greater efficiency. Nisin in combination with lactic acid (1.5%, 25 °C) spraying reduced aerobic bacteria, coliforms, and *E. coli* more efficiently than bacteriocin alone [10]. On the other hand, the bioprotective cultures (*E. faecium* PCD71 and *L. fermentum* ACA-DC179) and the combined application of different class IIa bacteriocins were able to reduce the growth of *L. monocytogenes* in different meat product [37,38,39]. However, in turkey meat stored aerobically at 10 °C, the use of *L. plantarum* BFE5092, producer plantaricins EF, JK and N as bioprotective culture failed to effectively inhibit *L. monocytogenes* [40]. These results highlight the importance of carefully testing the effectiveness of bacteriocins in the food systems for which they are intended to be applied against the selected target and non-target bacteria. Furthermore, the outgrowth of surviving or resistant bacterial populations points out that the tested bacteriocins do not ensure full inhibition of *L. monocytogenes* in a food product if not applied in combination with additional preservative measures.

### 4.2. Fermented Meat Products

Fermented meat products involve ecological determinants that strongly influence the establishment of a specific microbial association, this involving *L. sakei*, *L. curvatus*, and staphylococci coagulase positive cocci (*Staphylococcus* and *Micrococcus*) for acid production and nitrate reduction. Even when dry fermented sausage processing conditions, curing additives, and the presence of LAB starter cultures act as effective hurdles for pathogen control, they are not sufficient to prevent the survival of *E. coli* O157:H7 and *L. monocytogenes* during the manufacturing process. The use of bacteriocinogenic strains as functional starter cultures to reduce the risk of pathogen growth during meat fermentation has been suggested as an extra hurdle to ensure product safety and quality. In fermented sausages, the performance of bacteriocinogenic LAB as starters or co-cultures will depend on the processing technology, the presence of nitrate/nitrite, NaCl, and spices [41]. From a technological point of view, in order to eliminate both *E. coli* O157:H7 and *L. monocytogenes* during fermented sausage manufacturing, the goal would be the use of a functional starter culture that produces bacteriocins and causes a rapid pH decrease or a bacteriocinogenic strain in co-culture with an acidogenic LAB [42,43,44,45,46].

The bacteriocin-producing starter cultures involving *L. plantarum* and *L. curvatus* capable of controlling the growth of pathogenic microorganisms such as *L. monocytogenes* in fermented sausages and salami are shown in Table 1. The sakacin K producer *L. sakei* CTC494, used as a co-culture in addition to a commercial starter in different types of dry-fermented sausages, displayed high antilisterial effectiveness [47]. In addition, when sakacin producer *L. sakei* C2 as a starter culture was added to fermented sausage, effective control of spoilage and pathogenic microorganisms was achieved [48]. Because of their intrinsic competitiveness during sausage fermentation, *L. plantarum*, *L. sakei*, and *L. curvatus* are commonly used as meat starter cultures. Thus, the selection of class IIa bacteriocin-producing strains as functional starter cultures would be a useful alternative for protection against food-borne pathogens in fermented sausages; the results are dependent on sausage recipes, initial inoculation levels, and antilisterial starter applied [47]. However, the bacteriocinogenic *L. pentosus* 31-1 from traditional Chinese fermented ham was used both as a single starter and in co-culture during sausage manufacture, *L. innocua* and *S. aureus* were significantly reduced during the ripening process [49]. Likewise, the effect of a multiple starter involving the pediocin-producing *Pediococcus (P.) acidilactici* U318 and *L. plantarum* U201 during the fermentation of Balinese sausages allowed the elimination of *Enterobacteriaceae* [50]. More recently, *P. acidilactici* MCH14, pediocin PA-1 producer exhibited high effectiveness to inhibit *L. monocytogenes* and *Cl. perfringens* in Spanish dry-fermented sausages while *P. pentosaceus* BCC3772 (pediocin PA-1/AcH producer) was able to exert antilisterial activity during the fermentation of Nham, a Thai traditional fermented pork sausage, without significant changes in sensory characteristics [51,52]. Even when lactococcal bacteriocins seem to be not particularly adapted to sausage technology, in situ production of bacteriocin by *Lc. lactis* M against *L. monocytogenes* during fermentation and storage of mildly acid sausages showed high reduction of this pathogen [53].

On the other hand, the application of enterocins producing enterococci and/or their purified metabolites, as extra hurdles for preservation in sausage fermentation, can be beneficial, preventing the outgrowth of *L. monocytogenes*, *Clostridium*, and other spoilage LAB [54]. The bacteriocin-producing *E. casseliflavus* IM416K1 from Italian “cacciatore” sausage showed a high effectiveness for *L. monocytogenes* elimination, while *E. faecium* CTC492, an enterocin A and B producer, was unable to exert any positive antilisterial effect on fermented sausages compared to the batches treated with enterocins A and B, this being attributed to a high inhibition of the producer strain by refrigeration and the sausages’ ingredients [55,56]. However, the control of *L. monocytogenes* CECT4032 in sausages by adding the enterocin AS-48 producer strains *E. faecalis* A-48-32 and *E. faecium* S-32-81 as well as the semi-purified AS-48 bacteriocin to model sausages was described [57]. The use of enterococci in fermented meat is considered technologically unacceptable, and there is controversy over considering them as GRAS microorganisms. In meat-fermented products, enterococci, especially *E. faecium*, represent one of the LAB species that can be found in relatively high numbers and they may contribute, together with lactobacilli, to fermentation, conferring flavor to products by metabolic activities [54]. Among the features of technological relevance that bacteriocinogenic strains should meet are a good capacity for food colonization and bacteriocin production in the food matrix, where other preservative agents such as nitrites, sodium chloride, organic acids, pepper, and thermal treatments may also occur [57]. However, the entrapment of the bacteriocin-producing *L. curvatus* MBSa2 in calcium alginate caused a similar reduction of *L. monocytogenes* to the free strain in salami during the manufacturing process [48].

### 4.3. Chilled Vacuum and Modified Atmosphere Packaged Raw Meat

After slaughter and dressing, bacterial growth will depend on the storage conditions. Since temperature is the most important factor affecting the microbiota of meat, cooling of carcasses would be the first hurdle that spoilage and pathogen bacteria have to overcome. After carcasses dressing, the microbiota involves mesophilic and psychrotrophic bacteria, which are gradually selected during the chilling of meat; mesophile growth will no longer occur, while the latter organisms will predominate. Since pathogens are mostly mesophiles, chilled meat obtained in good hygienic conditions would not be expected to pose a sanitary risk. However, meat is a selective agent for aerobic microbiota, and a consortium of bacteria commonly dominated by *Pseudomonas* spp. is responsible for the spoilage of meat stored aerobically at temperatures between −1 and 25 °C [58]. In addition to chilling storage of meat, other environmental factors such as a gaseous atmosphere will also select bacterial growth towards CO_2_-tolerant organisms. Systems for retail meat distribution and commercialization are mainly based on vacuum-packaging (VP) or modified-atmosphere packaging (MAP) of meat cuts using low gas permeability films and refrigeration. Under these conditions, emergent psychotrophic facultative anaerobic and anaerobic bacteria such as *L. monocytogenes*, *Enterobacteriaceae*, *B. thermosphacta, Clostridium*, and deteriorative LAB can grow and cause different types of spoilage [58,59]. *B. thermosphacta* is a meat-borne microorganism that causes spoilage of refrigerated meat in aerobiosis and anaerobiosis, being less offensive in the last condition, in which glucose is mainly metabolized to lactic acid and ethanol [60]. The presence of fermentative LAB in low-oxygen environments is responsible for the production of lactic acid, acetic acid, and ethanol as major products; the deterioration caused is not regarded as particularly undesirable since the odor of the volatile fatty acids produced by these microorganisms disappears after opening the package [61]. Under restriction of oxygen, LAB are also able to produce H_2_S from cysteine, which oxidizes myoglobin to metmyoglobin, giving a green color to meat, whereas heterofermentative LAB in addition to lactic acid and CO_2_, during prolonged storage in a modified atmosphere produces fermentation products such as butyric acid and ethanol, reducing the shelf-life of meat. However, it is known that some other genera, such as *Clostridium* and *Enterobacteriaceae*, can multiply in VP meat, causing deterioration (proteolysis, loss of texture and hydrogen sulfide accumulation) and pack blowing at refrigeration temperatures [59]. *Clostridium* species are able to produce blowing of chilled vacuum packages, while the presence of *Enterobacteriaceae* is of particular importance, for its high deteriorating potential and food safety concerns because of some pathogenic species [59].

During VP of raw meat, an additional hurdle to reduce the risk of deteriorative bacteria and pathogens growth would be the use of competitive bacteriocin-producing LAB and their bacteriocins for shelf-life extension of raw meat and poultry. Given that, among the main LABs isolated from chilled VP or MAP meats, different species belonging to *Lactobacillus, Leuconostoc, Carnobacterium*, and *Weissella* were often identified [62], it is expected that strains from these genera would be more adapted and likely to be applied as protective cultures. From an application point of view, the use of bacteriocinogenic LAB strains as bioprotective cultures for commercial meat preservation would be more feasible, since nisin is the only bacteriocin approved in foods. Table 1 shows efficient antimicrobial strategies in the control of pathogens commonly found in chilled vacuum and modified atmosphere packaged raw meat [63,64,65,66,67,68].

### 4.4. Cooked Ready to Eat Meat Products

Cooked, cured or not, meat products are economically important ready to eat (RTE) commodities, including cooked ham, emulsion-style sausages, pork loin (smoked or not), bacon, pâté, and cooked turkey/poultry products. In these products, vegetative cells are killed by heat treatment (65–75 °C), with post-processing recontamination determining their shelf life [69]. Cooked meat products’ composition and handling after thermal treatment, instabilities within the cold chain, processing line and storage hygienic conditions, and domestic mishandling practices mostly determine the microbial composition [70,71]. Spoilage of these meat ecosystems involves acid production, color deterioration, and off-flavor compounds that often lead to product rejection. Under these conditions, *Lactobacillus*, *Carnobacterium*, *Leuconostoc*, *Weissella*, and *B. thermosphacta* become the dominant LABs in cooked meat ecosystems [60,69,72]. In addition to the presence of spoilage organisms, the psychotropic pathogen *L. monocytogenes* may also be present, mainly because of post-processing contamination. The presence of this opportunistic pathogen in the food chain is of greatest concern to the RTE food industry as there is no cooking or other microbial inactivation step between production and consumption. Although a high prevalence of *L. monocytogenes* was reported from soft/fresh cheeses and smoked fish [1,2], RTE and delicatessen meat and poultry products were described as a very important source [71,73,74]. As *L. monocytogenes* is widely distributed in the environment, it is likely that many contaminated RTE-meat products may have acquired the pathogen via routes other than the beef production chain. The relative prevalence of this pathogen obtained over multiple surveys in beef, pork, and poultry from the farm to the final RTE products point out that antimicrobial interventions to eliminate *L. monocytogenes* must be applied. A number of studies have reported on the use of bacteriocinogenic LAB strains to extend the shelf-life of cooked meat products. Table 1 shows some of these reports including strains of *Lactocillus* and *Leuconostoc* species as biopreservatives [75,76,77,78,79]. However, although meat-borne *Leuconostoc* species were highly effective at preventing pathogen and contaminant growth in RTE products, this LAB may also be responsible for greening and slime formation in cooked bacon; the use of *Lc. lactis* and *L. sakei* as biopresevative cultures was able to inhibit the growth of *Leuconostoc (Ln.) mesenteroides*, reducing the spoilage risk [80]. Moreover, the inhibition of *L. monocytogenes* on hot dogs using bacteriocin mixtures based on their different mode of action was recently reported [81]. It is generally assumed that a mixture containing more than one bacteriocin would be bactericidal to more cells in a sensitive population, since cells resistant to one bacteriocin would be killed by another. In addition, the use of culture extracts provides an opportunity for the biological activity to be standardized, whereas the use of live cultures to provide antimicrobial protection in non-actively growing situations is weak. Bacteriocin extracts produced by food-grade LAB could be freely applied as food ingredients to act as biopreservatives in RTE meats and other products where they had been proven effective against targeted pathogens and/or spoilage organisms [81].

## 5. Other Antimicrobial Interventions

### 5.1. Antimicrobial Packaging Systems

Due to increased demand for greater stringency directed to hygiene and safety issues associated with fresh and processed meat products, together with ever-increasing demands by retailers for extension of product shelf-life and the requirement to meet consumer expectations in relation to convenience and quality, the food packaging industry has made important progress in recent years. Active packaging refers to the incorporation of certain additives into packaging systems—whether loose within the pack, attached to the inside of packaging materials, or incorporated within the packaging materials themselves—with the aim of maintaining or extending product quality and shelf life. Packaging may be termed active when it performs some desired role such as extending shelf-life or improving safety or sensory properties, other than providing an inert barrier to external conditions. Active packaging systems include oxygen scavengers, carbon dioxide scavengers and emitters, moisture control agents, and antimicrobial packaging technologies [82].

Antimicrobial active packaging has been the subject of experiments because it is believed to have significant potential for improving food safety and prolonging the shelf-life of food products [83,84]. Many preservatives, such as organic acids and their salts, plant extracts, silver-substituted zeolite, lysozyme, and chlorine dioxide, have been successfully incorporated in packaging materials to confer antimicrobial activity to food packaging [85]. To control undesirable microorganisms on foods, antimicrobial substances can be incorporated in food packaging film structures, synthetic polymers, and edible films, as well as coating bioactive agents on the surface of the packaging material or utilizing antimicrobial macromolecules with film-forming properties or edible matrices [83]. There is a growing interest in edible coatings due to factors such as environmental concerns, new storage techniques, and market development for underutilized agricultural commodities [85]. Edible coatings and films prepared from polysaccharides, proteins, and lipids have a variety of advantages such as biodegradability, edibility, biocompatibility, appearance, and barrier properties [84]. Biopolymers such as alginate, chitosan, and gelatin can be used for the packaging of food products with the advantage over synthetic polymers that they are biodegradable and renewable as well as edible [85]. However, many studies have focused on synthetic polymeric (inedible) packaging materials, especially for polyethylene (PE), polypropylene (PP) polyvinyl chloride (PVC), polyvinyilidene chloride (PVDC), vinyl acetate ethylene (VAE), ethylene acrylic acid (EAC), linear low-density polyethylene (LLDPE), low-density polyethylene (LDPE), and multilayer films, these being commonly used as an inner layer in packaging combinations. Due to the disadvantages of high temperatures and shearing associated with the extrusion process of these polymers, an alternative to the incorporation of antimicrobial compounds during extrusion is to apply them as a coating. This procedure has the advantage of placing the specific antimicrobial additive in a controlled manner without subjecting it to high temperature or shearing forces. The coating can serve as a carrier for antimicrobial compounds in order to maintain high concentrations of preservatives on the surface of foods [83]. The antimicrobial packaging film must contact the surface of the food so that the antimicrobial compound can diffuse to the surface; its gradual release from a packaging film to the food surface may have an advantage over dipping and spraying foods. In the latter processes, antimicrobial activity may be lost or reduced due to inactivation of antimicrobials (particularly bacteriocins) by food components or dilution below the active concentration due to migration into the foods [86]. The incorporation of the active biological agent in the packaging material was accomplished by different methods such as pouch contact, soaking, impregnation, and contact. It was reported that soaking and contact procedures demonstrated higher efficiency than pouch-contact to incorporate bacteriocins produced by *L. curvatus* CRL705 on a polyethylene (PE)-based film, allowing homogeneous and confined inhibition areas when inhibitory effectiveness was assayed into the agar [87]. However, soaking, applied to develop active PE-oriented polyamide films with an antilisterial bacteriocin from *L. curvatus* 32Y, was unable to obtain uniform bacteriocin diffusion from the film into the agar [88].

Due to their GRAS status, LAB inhibitory metabolites in packaging materials are promising, particularly for meat and meat products. Bacteriocins produced by LAB are cationic, hydrophobic, and amphiphilic peptides, with a broad spectrum of antimicrobial activity, mainly against Gram-positive bacteria; among them, *L. monocyotgenes* is one of the most important target microorganisms to be controlled, mostly in processed and RTE foods. Since nisin is the only bacteriocin approved as an additive for meat products, many research groups have developed antimicrobial solutions for meat packaging containing this bacteriocin in order to delay or suppress the growth of foodborne spoilage microorganisms and pathogens [83]. Indeed, nisin’s small molecular size allows the production of films that release the peptide after contact with food. Nisin is commonly incorporated into coatings, together with acids, and only occasionally with other compounds such as EDTA when antimicrobial action against Gram-negative bacteria was desired. The combination of bioactive packaging with refrigeration and storage in a vacuum/modified atmosphere would supply food products with improved hygienic qualities. Studies on the utilization of nisin in edible and inedible films for the preservation of meat and meat products showed reductions in the range of 3 log cycles and below [82], although studies done in liquid media have reported log-reductions between 6 and 9 by using nisin and chitosan in the coating [89]. Nisin application to control *L. monocytogenes*, *B. thermosphacta*, *Enterobacteriaceae*, and spoilage LAB included its combination with PE, LDPE, nisin/EDTA-coated co-extruded plastic barrier, PE-based plastics, cellophane, chitosan, and soy protein isolate (SPI)/essential oils for raw beef, fresh ham steak, beef chuck tender slices, ground beef, and ground-beef patties; polyolifine, packaging paper inserts, and alginate/corn zein/polyvinyl alcohol (PVA) for cooked sliced ham and turkey breast; cellulose casings, corn zein/propylene glycol/ethanol, wheat gluten, and SPI for chicken, turkey bologna, and beef/turkey frankfurters; as well as pectin and gelatin/sodium alginate and PVA for pork/convenience fermented sausage [90,91,92,93,94]. In addition to nisin, much research has been done on the development of antimicrobial films using other semi-purified bacteriocins. Pediocins (freeze-dried powder and ALTA^®^) incorporated in cellulose-based materials were used to inhibit the growth of *L. monocytogenes* on sliced ham, turkey, and beef [95,96]. Concentrated enterocin 416K1 and semi-purified enterocins A and B, produced by *E. casseliflavus* IM416K1 and *E. faecium* CTC492 used to activate an organic–inorganic hybrid coating applied to a LDPE and alginate/zein/PVA films, respectively, were reported to be highly effective at inhibiting *L. monocytogenes* in contaminated frankfurters and cooked ham stored at refrigeration temperatures [97,98]. The efficacy of antimicrobial films obtained by the activation of PE, multilayer and wheat gluten films with the bacteriocins produced by *L. curvatus* 32Y and CRL705 to control *Listeria* growth in pork frankfurters and wieners, respectively, was also reported [99,100]. Pullulan films containing sakacin A produced by *L. sakei* also proved to be effective against *L. monocytogenes* growth in turkey breast during three weeks of refrigerated storage [101]. In addition, a novel bioactive packaging based on the incorporation of the bacteriocinogenic *L. sakei* LQC1089 cells into sodium–caseinate films for the control of *L. monocytogenes* in fresh beef showed significant inhibition of the pathogen [102]. Nevertheless, acids and their salts were also employed as antimicrobials, combined with film materials. Acids and salts are generally those already in use for food preservation or acidification and include sorbic acid, potassium sorbate, and p-amino benzoic acid. These chemicals, besides being used as single antimicrobials, were also used in combination with matrices such as chitosan, whey/soy protein isolates, corn zein, cellulose, and methylcellulose as well as PE, LDPE, PVC, and PVDC (saran) to control contaminants and pathogens including LABs, *Enterobacteriaceae, Salmonella*, and *L. monocytogenes* in different meat products.

### 5.2. Pathogen Biofilms Prevention

Biofilms can form anywhere as long as nutrients and a surface for adhesion are present. Industrial systems use them in wastewater management, brewing, pulp and paper manufacturing, as well as food processing. Microorganisms in biofilms live in a self-produced matrix of hydrated extracellular polymeric substances (EPS) that form their immediate environment. EPS are mainly polysaccharides, proteins, nucleic acids, and lipids; they provide the mechanical stability of biofilms, mediate their adhesion to surfaces, and form a cohesive, three-dimensional polymer network that interconnects and transiently immobilizes biofilm cells [103]. It is known that attached microorganisms are more resistant than free-living cells to sanitizing compounds, thus if the remaining viable cells are transferred to the food, they could have a serious negative impact on the storage quality and safety of that food. Infections caused by food-associated pathogens capable of forming biofilms such as *L. monocytogenes*, *Campylobacte*r spp., and *Salmonella* spp. seriously affect public health on a global scale with high annual healthcare costs [104]. Controlling the adhesion of microorganisms to food contact surfaces is an essential step in meeting the goals of food processing. The settlement and persistence of foodborne pathogens in food processing environments is intimately related to their response to both biotic and abiotic factors. Most of the pathogens involved in foodborne diseases are able to adhere and form biofilms on most materials used and under almost all the environmental conditions encountered in food production plants [105]. In particular, *L. monocytogenes* is capable of adhering and forming biofilms on food-contact surfaces such as polystyrene (PS), polytetrafluoroethylene (PTFE) glass, and stainless steel (SS) and persisting for long periods [106]. The major problem with *L. monocytogenes* biofilms, particularly at slaughter and meat processing plants, and on equipment surfaces, is their persistent resistance to desiccation, UV and light, and treatments with antimicrobial and sanitizing agents [74]. Biofilm prevention and control is therefore a priority in the food industry; current trends for naturally controlling the shelf life and safety of foods include the use of chemicals and biological agents. Because of the greater resistance of microorganisms within biofilms compared to their planktonic counterparts, the use of disinfectants and biocides is not enough to remove the biofilms. The ability of *L. monocytogenes* to colonize different surfaces at low temperatures of food processing and storing, together with the fact that this pathogen survives and grows in biofilms at 2 to 4 °C, increases its prevalence; contamination by this pathogen of equipment and surfaces in retail meat areas, largely during slicing and packaging after cooking, has been widely described [31,107,108]. The development of innovative strategies for *Listeria* control in food and food processing environments constitutes a major challenge.

LABs were reported as good candidates to settle protective biofilms on food industry surfaces to control *L. monocytogenes* colonization. The modification of the physicochemical properties of the solid surfaces, the competition for nutrients, and/or the production of antimicrobial compounds are among the evaluated approaches for pathogen inhibition. Bacteriocinogenic LAB settlement on surfaces showed advantages over simply conditioning the surface with bacteriocins by limiting nutrient supply and competitive inhibition. The bacteriocinogenic *Lc. lactis* UQ2, as well as its spray-dried crude bacteriocin fermentate, were shown to reduce by more than 5 log the planktonic and sessile cells of *L. monocytogenes* Scott A attached to SS chips [109]. Similarly, the meat-borne *L. sakei* 1 and its bacteriocin sakacin 1 were able to inhibit early biofilm formation by *L. monocytogenes* on SS surface [110]. Recently, the meat-borne *L. sakei* CRL1862, a curvacin A producer, was highly competent to form biofilm on meat industry relevant SS and PTFE surfaces [111]. When its ability to displace, exclude, and compete *L. monocytogene*s biofilm formation on these industrial surfaces was evaluated, an effective pathogen inhibition through the three assayed strategies was reported, inhibition being more efficient on PTFE than on an SS surface [112]. Similarly, high antilisterial efficacy was achieved by pre-established biofilms of bacteriocin–producing LABs used as biosanitizers. Biofilm settlements of *L. plantarum* 35d in a small-scale model, as well as pretreatment with an LAB (*P. acidilactici*, *L. amylovorus*, and *L. animalis*) cocktail on SS coupons cut from the blade of a deli slicer machine, directly competed with *L. monocytogenes* for attachment to surfaces with a significant pathogen exclusion [113,114]. The probiotic strains *L. paracasei* and *L. rhamnosus* were reported to effectively inhibit biofilm formation by *L. monocytogenes* through the mechanisms of competition, exclusion, and displacement [115]. Moreover, the biofilmogenic dairy *L. pentosus* LB3F2 was able to strongly hamper the adhesion of *S. aureus* on abiotic surfaces such as PS and SS slides [116].

Nevertheless, recent efforts focusing on targeting the molecular determinants regulating biofilm formation have been performed. In particular, an interesting strategy targets quorum sensing (QS) systems, which regulate gene expression in response to fluctuations in cell population density governing essential cellular processes including biofilm formation. Strategies that target QS, in contrast to bactericidal approaches, exert less selective pressure for resistance development to inhibitory agents. The disruption of QS signaling, also termed quorum quenching (QQ), refers to the inhibition of QS through degradation and/or inactivation of the QS signaling molecules [104]. Prevention of biofilm formation targeting QS system is based on inhibition of cell-to-cell communication, this being performed in a number of ways including inhibition of signaling peptides synthesis or degradation, prevention of signaling peptide-receptor binding, or inhibition of the signal transduction cascade [117]. As an anti-biofilm tool, QQ is based on the addition of inhibitory molecules (or the producer itself) as a bioagent in the food industry or into an antibacterial formulation for clinical use. Many organisms produce QQ molecules when competing with neighboring species for nutrients and space. Besides plant extracts and bacterial enzymes acting as QQ in foods [104], bacteriocins produced by LAB may act as QQ molecules. Several studies explored the relationship between biofilm prevention and QS inhibition using bacterial antimicrobial peptides has recently been reported. However, for clinical use, *L. reuteri* RC-14 (human vaginal isolate) was able to inhibit the *S. aureus* QS system *agr*, by the production of two cyclic dipeptides involved in the interspecies communication [118]. Similarly, subtilosin, a cyclic lantibiotic produced by *Bacillus subtilis*, inhibited *Gardnerella vaginalis* biofilm by downregulation of QS-associated gene expression [119]. Anti-QS or QQ-mediate approaches for the control of pathogens biofilm on food industrial premises would represent a safe and environmentally friendly sanitation method to mitigate post-processing food contamination.

## 6. Antimicrobial Hurdle Combinations

Antimicrobial intervention technologies through implementation of decontamination treatments or antimicrobial procedures for inhibition or reduction of microbial growth are gaining interest in order to reduce bacterial contamination. These approaches must be safe, economical, and feasible in the production process, maintaining the organoleptic characteristics and consumers’ acceptance. The concept of hurdle technology began to apply in the food industry in a rational way after the observation that the survival of microorganisms greatly decreased when they were challenged with multiple antimicrobial factors. After exposure of a bacterial population to a single antimicrobial intervention there is often a heterogeneous response, depending on the intensity of treatment as well as many other factors; a fraction of the population may receive a lethal dose of the antimicrobial treatment, leading to cell death, while sub-lethally injured and/or resistant cells may repair the damage and survive [11]. By contrast, when a combination of antimicrobial factors is applied, multiple cell damages will be produced, whereby the cell incurs much higher energy costs, leading to exhaustion and cell death. Therefore, survival and proliferation probabilities for cells exposed to multiples hurdles are very low, allowing the use of lower treatment doses compared to their individual application. In this context, the use of sequential interventions at different points during meat and meat product processing (multiple hurdle approach) must be considered in order to enhance the microbiological safety of meat and poultry and their derived products. Examples of hurdle combinations involving LAB metabolites, applied to the different meat and poultry ecosystems, are shown in Table 2 and Table 3.

**Beef and poultry carcasses**. Despite efforts targeted at the maintenance of good hygiene practice during meat production, the prevention of carcass contamination with meat-borne pathogens during slaughter is difficult. In meat carcasses, application of interventions at slaughter plants reduced the bacterial loads on cattle hides and beef carcasses to some extent, with *Enterobacteriaceae*, *Salmonella*, and *E. coli* O157:H7 being of special interest. TCombinations of decontamination treatments involving physical (hot water, steam, dry heat, air chilling, steam vacuuming, and irradiation), chemical (chlorine, cetylpyridinium chloride, alcohols, H_2_O_2_, ozone, sodium hydroxide, trisodium phosphate (TSP), and organic acids), and biological (bacteriophages and bacteriocins) approaches are known to yield more promising results. Thus, the combined effect of hot water, steam, acetic acid, and/or lactic acid mainly yielded reductions for several bacterial species below two orders of magnitude on beef carcasses. With regard to poultry, *Campylobacter* is of special importance as poultry meat is often implicated as a risk factor; different combinations of antimicrobial interventions were assayed for the decontamination of poultry carcasses and parts (Table 2). Amongst the combinations of chemical and physical interventions, the combination of hot water with TSP or sodium carbonate, spraying with acetic acid, or TSP followed by immersion in chlorinated water, as well as levulinic acid plus sodium dodecyl sulfate on inoculated chicken wings were more efficient than single treatments. Most of the evaluated studies addressed the efficacy of chemical treatments, followed by physical interventions. In particular, hot water, steam, electrolyzed water, and irradiation proved to be effective in reducing the bacterial load on poultry carcasses, ranging between 0.9 to 3.9 log cycles, with steam being the most effective [32]. However, nisin, as the only commercially approved bacteriocin, has been used in combination with hot water and lactic acid to decontaminate red meat carcasses [120]. In addition, the reduction or elimination of *Salmonella*, *E. coli*, and particularly *E. coli* O157:H7 level in beef hides and carcasses was also achieved by the combined action of lactic and acetic acids spraying as well as NaOH and lactic and acetic acids [121,122].

**Raw meats, fermented, cooked and RTE meat products**. Low temperatures, VP, and MAP storage of meat cuts using low gas permeability films represent initial hurdles applied to meat to extend its shelf-life, as previously described. However, these preliminary hurdles can be applied in combination with additional chemical, physical (such as high hydrostatic pressure (HHP), irradiation, and pulsed electric fields), and biological treatments involving bacteriocins and bacteriophages for pathogen inactivation in meat, poultry, and derived products (Table 2). To increase their efficacy against vegetative cells, a combination of these preservation technologies under the so-called hurdle concept has also been investigated to preserve fresh meat. A combination of organic acids in water as dipping solutions was applied for chemical decontamination of chicken skin and meat, resulting in substantial reductions [123]. Recently, the application of lauric arginate plus a vinegar solution via a commercial spray cabinet to inactivate *S. thiphimurium* on skinless chicken meat was reported as a viable treatment [124]. Moreover, higher inhibition efficacy was reported when the combined strategy of chemical preservatives, non-thermal technologies, and bacteriocins and/or bacteriocinogenic LAB strains was evaluated. Nisin-based treatments containing variable concentrations of citric acid, EDTA, and Tween 20 were used to inhibit *S. thyphimurium* on broiler drumstick skin and whole drumsticks to extend shelf-life, while the effective inhibition of *L. monocytogenes* using nisin and NaCl combination on raw buffalo meat mince was also reported [125,126]. Similarly, nisin combined with oregano essential oil, organic acids salts, and cinnamon was reported to be effective against *S. enteritidis* and *L. monocytogenes* in minced sheep meat and a sausage model [127,128]. In addition, although the single application of enterocin AS-48 was very effective to control *L. monocytogenes*, *L. sakei*, *B. thermosphacta*, and *S. carnosus* spoilage in a cooked ham model under VP/MAP conditions at 5 °C, enhanced AS-48 activity in combination with chemical preservatives was reported [79,80]. Similarly, the antimicrobial activity of three LAB strains (Lactiguard™) and the cell-free extract of these LAB on *L. monocytogenes* inoculated onto frankfurters containing lactate/diacetate was highly effective at reducing pathogen growth after eight weeks of refrigerated storage [81]. On the other hand, bactericidal synergisms, through non-thermal technologies such as HHP and irradiation, in combination with other antimicrobials, was shown to be highly effective at preventing pathogen growth in meat and meat products. Indeed, bacteriocins (nisin, enterocins A and B, sakacin K, and pediocin AcH), and HHP treatment in a meat model exhibited greater inactivation of *E. coli* while maintaining slime-producing LAB and *L. monocytogenes* below the detection level during chilling storage [129], with antimicrobial interventions increasing with decreasing storage temperature. Similarly, the combination of HHP technology with bacteriocinogenic LAB strains, nisin, enterocins A and B, lactate salts, and glucono-delta-lactone for the inhibition/reduction of aerobic mesophiles and psychrophiles, *L. monocytogenes* and *Salmonella* on mechanically recovered poultry meat, dry cured hams and RTE cooked hams was more effective than the use of nisin as the only antimicrobial approach [130,131,132]. Moreover, the combined action of biodegradable packaging activated with enterocins A and B with HHP as post-processing treatment was able to inhibit *L. monocytogenes* during storage of RTE cooked ham [133]. In addition, irradiation as another non-thermal technology was applied in combination with organic acids and/or their salts to control *L. monocytogenes* in RTE meat products such as turkey, ham, and frankfurters [134,135]. On the other hand, bacteriophages should also be considered in hurdle technology combined with different preservation methods such as nisin and HHP. Their use to control the growth of pathogens such as *Salmonella, L. monocytogenes, S. aureus,* and *Campylobacter jejuni*, as well as *E. coli* O157:H7 in a variety of refrigerated foods such as poultry and ground beef, has been reported [136,137,138].

## 7. Conclusions

Microbial antagonism has great potential to improve safety in the food chain and thus reduce associated public health risks. Research on the biocontrol of pathogenic bacteria through the meat food chain has provided a wide array of treatment options based on different antimicrobial interventions involving a combination hurdles. Recently, the influence of food preservation methods on the physiology and behavior of microorganisms in foods, such as their homeostasis, metabolic exhaustion, and stress reactions, was taken into account and a novel concept of multi-target food preservation has emerged. As shown in this review, many bacteriocins and bacteriocinogenic strains perform satisfactorily in different meat ecological systems. The combination of physical and chemical methods was highly effective to extend the shelf-life of raw/fresh red meats and poultry, while biological approaches and their combinations allowed for the biopreservation of meat-derived products, mostly RTE foods. In this context, the range of natural antimicrobial molecules produced by LAB, combined with non-thermal technologies, can be used in food preservation, offering a satisfactory approach to solve many of our current food-related issues.

## Figures and Tables

**Table 1:** Antimicrobial interventions of LAB and their metabolites on different meat products.

	LAB and Antimicrobial Metabolites	Microorganism Inhibition	Meat Product	Reference
I Meat conditioning and raw meats	Acetic, citric and lactic acids and their salts	Aerobic bacteria and Enterobacteriaceae	In fresh pre-eviscerated carcasses	[33]
	Acetic and lactic acid spraying	Coliforms, Enterobacteriaceae and *E. coli*	Carcasses	[34,35]
	Bacteriophages	*L. monocytogenes*	Carcasses (decontamination limited)	[36]
	Nisina	Marginal reductions of *Listeria*	Beef carcasses yielded	[10]
	Nisin and lactic acid spraying	Aerobic bacteria, coliforms and *E. coli*	Beef carcasses yielded	[10]
	*E. faecium* PCD71 and *L. fermentum* ACA-DC179	*L. monocytogenes*, *S. enteritidis*	Raw ground chicken meat	[37]
	*L. sakei* CWBI-B1365 and *L. curvatus* CWBI-B28	*L. monocytogenes*	Raw beef and poultry meat	[38]
	sakacin A produced by *L. sakei* IDE0216	*L. monocytogenes*	Ground beef	[39]
	*L. plantarum* BFE5092 (plantaricins EF, JK and N producer)	*L. monocytogenes*	Turkey meat	[40]
Fermented meat products	*L. plantarum* and *L. curvatus (*plantaricin 423 and curvacin DF126 producer)	*L. monocytogenes*	Ostrich meat salami	[42]
	*L. plantarum* EC52 bacteriocinogenic strain	*L. monocytogenes* and *E. coli* O157:H7	Meat sausage	[43]
	*L. curvatus* DF38 (curvacin DF38 producer)	*L. monocytogenes*	Different meat origins salami	[44]
	*L. curvatus* 54M16 (sakacin producer)	*L. monocytogenes*	Fermented sausage	[45]
	Sakacin P and sakacin X produced by *L. curvatus*	*L. monocytogenes*	Brazilian salami	[46]
	Sakacin K produced by *L. sakei* CTC494	*L. monocytogenes*	Dry-fermented sausages	[47]
	Sakacin produced by *L. sakei* C2	Spoilage and pathogenic microorganisms	Sausage	[48]
	*L. pentosus* 31-1 bacteriocinogenic strain	*L. innocua* and *S. aureus*	Sausage	[49]
	Pediocin-producing Pediococcus (P.) acidilactici U318 and *L. plantarum* U201	Enterobacteriaceae	Balinese sausages	[50]
	*P. acidilactici* MCH14 (pediocin PA-1 producer)	*L. monocytogenes* and *Cl. perfringens*	Spanish dry-fermented sausages	[51]
	P. pentosaceus BCC3772 (pediocin PA-1/AcH producer)	*Listeria* strains	Nham, a Thai traditional fermented pork sausage	[52]
	Bacteriocins produced by *Lc. Lactis* M	*L. monocytogenes*	Mildly acid sausages	[53]
	Enterocins producing enterococci	*L. monocytogenes* and other spoilage LAB	Sausage	[54]
	Bacteriocin-producing *E. casseliflavus* IM416K1	*L. monocytogenes*	Italian “cacciatore” sausage	[55]
	*E. faecium* CTC492 (enterocin A and B producer)	*L. monocytogenes*	Italian “cacciatore” sausage	[56]
	*E. faecalis* A-48-32 and *E. faecium* S-32-81 and semipurified AS-48 bacteriocin	*L. monocytogenes* CECT4032	Sausages	[57]
	Bacteriocin-producing *L. curvatus* MBSa2 in calcium alginate	*L. monocytogenes*	Salami	[48]
Chilled vacuum- and modified atmosphere-packaged raw meat	*L. curvatus* CRL705 and lactocin AL705	*Listeria* and *B. thermosphacta*	VP fresh beef	[23,63]
	Sakacin P/sakacin P-producing *L. sakei* Lb790 and sakacin K/sakacin K-producing *L. sakei* CTC494	Listeriostatic effect	VP chicken cold cuts and refrigerated VP fresh meat	[64,65]
	Bacteriocin-producing *L. sakei* CECT4808	*B. thermosphacta* and spoilage LAB	VP sliced beef	[66]
	Bacteriocinogenic strans of *L. sakei*	Anaerobic microbial population	VP lamb	[67]
	Lactocin 705, enterocins A/B and sakacin P	*Listeria* and B. thermosphacta	VP raw beef and chicken	[23,55,64,65]
	Nisin or nisin/EDTA	*B. thermosphacta*	VP fresh beef	[68]
Cooked ready to eat meat products	*L. sakei* 1 and 10A	*L. monocytogenes*	VP baked ham	[75]
	*Ln. Carnosum* 3M42	Spoilage microorganisms	Artisan-type cooked ham	[76]
	*Ln. Carnosum* 4010	*L. monocytogenes*	VP sliced cooked pork product	[77]
	*Ln. mesenteroides* L124 and *L. curvatus* L442	*Listeria* strains	Sliced cooked cured pork	[78]
	Bacteriocins produced by *Ln. mesenteroides* L124 and *L. curvatus* L442	*Listeria* strains and *B. thermosphacta*	Sliced cooked cured pork	[79]
	Pediocin PA-1 produced by *P. acidilactici* MCH14	*L. monocytogenes* and *Cl. perfringens*	Frankfurters	[51]
	*Lc. Lactis* and *L. sakei*	*Ln. mesenteroides*	Cooked bacon	[80]
	Bacteriocins mixtures (different classes)	*L. monocytogenes*	Hotdogs	[81]

**Table 2:** Antimicrobial hurdle combinations applied to cattle hides, beef/poultry carcasses, and parts.

Combination	Microorganisms	Meat Product	Reference
Acetic acid + hot water	*E. coli* O157:H7 *Salmonella*	Beef carcasses and carcass parts	[34]
	Aerobic bacteria *E. coli* O157:H7 *Salmonella*	Cattle hides	[35]
Lactic acid + hot water	Aerobic bacteria Coliforms *S. thyphimurium*	Beef carcasses	[35]
	Aerobic bacteria *Enterobacteriaceae*	Pre-eviscerated beef carcasses	[33]
	*E. coli* O157:H7	Beef carcasses	[30]
Lactic acid + steam	*L. innocua*	Chicken skin	[139]
Lactic acid + nisin	Aerobic bacteria Coliforms *E. coli*	Red meat carcasses	[10]
Lactic acid + acetic acid spraying	*E. coli*	Beef carcasses	[122]
Lactic acid + acetic acid + NaOH	*E. coli* O157:H7 *Salmonella*	Beef hides	[121]
Acetic acid + air injection	Aerobic bacteria *Enterobacteriaceae*	Poultry carcasses	[32]
Acetic acid + ultrasound	Aerobic bacteria	Broiler drumsticks skin	[32]
Na_2_CO_3_ + hot water	*L monocytogenes S. aureus S. typhimurium*	Chicken wings	[32]
Electricity + chlorinated compounds	*S. thyphymurium*	Chicken skin	[32]
NaClO + electrolized water	Aerobic bacteria *Campylobacter jejuni E. Coli Salmonella*	Broiler carcasses	[140]
Nisin + EDTA + citric acid + Tween 20	*S. typhimurium*	Broiler carcasses	[125]
Nisin + hot water	*L. innocua*, *Carnobacterium divergens* *B. thermosphacta*	Beef carcasses tissue	[120]

**Table 3:** Antimicrobial hurdle combinations applied to raw meats, fermented sausages, and cooked/RTE meat products.

Combination	Microorganism	Meat Product	Reference
Lauric arginate + vinegar	Aerobic bacteria *S. thyphimurium*	Skinless chicken meat	[124]
Lactic acid + other chemicals + water	*Campylobacter jejuni*	Chicken skin and meat	[123]
Nisin + NaCl	*L. monocytogenes*	Raw buffalo meat mince	[126]
Nisin + oregano essential oil	*S. enteritidis*	Sheep meat mince	[127]
Nisin + organic acids + cinnamon	*L. monocytogenes*	Sausage model	[128]
HHP + nisin	*L. monocytogenes*	Dry cured ham	[131]
HHP + nisin + enterocins A/B + sakacin K + pediocin AcH	Slime-producer LAB *Salmonella Staphylococcus*	Meat model system	[129]
HHP + nisin+ lactate salts	*L. monocytogenes Salmonella*	Sliced cooked ham	[130]
HHP + enterocins A/B	*L. monocytogenes*	Cooked ham	[133]
HHP + nisin + glucono-δ-lactone	Aerobic bacteria	Mechanically recovered poultry meat	[141]
HHP + enterocins A/B activated packaging	*L. monocytogenes*	Cooked ham	[98]
HHP + antilisterial *W. viridescens*	*L. monocytogenes*	Cooked chicken mince	[132]
Irradiation + lactate/diacetate salts	*L. monocytogenes*	Frankfurters	[135]
Irradiation + lactate/diacetate/benzoate salts	*L. monocytogenes*	RTE turkey ham	[134]
Bacteriophage	*Campylobacter jejuni Campylobacter coli*	Broiler chickens	[136]
Bacteriophage	*Salmonella*	Chicken meat and skin	[137]
Bacteriophage	*E. coli* O157:H7	Ground beef	[138]
